# Impact of anti-T-lymphocyte globulin dosing on graft versus host disease in matched sibling peripheral blood stem cell transplantation

**DOI:** 10.1038/s41409-025-02761-5

**Published:** 2026-01-21

**Authors:** Radwan Massoud, Evgeny Klyuchnikov, Silke Heidenreich, Maroly Bohorquez Manjarres, Ina Rudolph, Rolf Krause, Gaby Zeck, Claudia Langebrake, Adrin Dadkhah, Rusudan Sabauri, Christian Niederwieser, Mathias Schäfersküpper, Franziska E. Marquard, Maraike Harfmann, Sofia Oechsler, Gunnar Weise, Kristin Rathje, Nico Gagelmann, Catherina Lueck, Normann Steiner, Christine Wolschke, Francis Ayuk, Nicolaus Kröger

**Affiliations:** Department of Stem Cell Transplantation, University Medical Center Hamburg-Eppendorf, Hamburg, Germany

**Keywords:** Drug development, Stem-cell therapies

## Abstract

Anti-T-lymphocyte globulin (ATLG) is commonly administered to reduce graft-versus-host disease (GVHD) in allogeneic stem cell transplantation (allo-SCT). However, the optimal ATLG dose in matched-sibling-donor (MSD) peripheral blood stem-cell transplantation (PBSCT) remains uncertain. We compared two ATLG doses (15 mg/kg vs. 30 mg/kg) in the MSD-PBSCT setting to assess allo-SCT outcomes. In this single-center retrospective study, we included 165 consecutive patients with hematologic malignancies who underwent MSD-PBSCT. Of these, 71 received 15 mg/kg ATLG (ATLG-15), and 94 received 30 mg/kg ATLG (ATLG-30). ATLG-15 was associated with earlier leukocyte (median 11 vs. 12 days, *p* = 0.004) and platelet engraftment (median 12 vs. 15 days, *p* = 0.0002). Moderate/severe chronic GVHD at 2 years was significantly higher with ATLG-15 (43% vs. 28%, *p* = 0.045), with no difference in OS, PFS, NRM, or CIR. In multivariable analysis, ATLG-30 was associated with improved GRFS (HR 0.47, *p* = 0.02). After propensity score matching, GRFS and all-grade cGVHD remained significantly better with ATLG-30 (*p* = 0.047), with a trend toward reduced moderate/severe cGVHD (*p* = 0.067). In AML/MDS patients not receiving TBI (*n* = 108), moderate/severe cGVHD remained lower with ATLG-30 (19% vs. 38%, *p* = 0.039). Our study suggests that ATLG-30 in MSD-PBSCT reduces moderate/severe cGVHD and improves GRFS.

## Introduction

Allogeneic stem cell transplantation is a potentially curative strategy for hematological malignancies due to the graft versus tumor effect [1, 2]. However, graft versus host disease (GVHD) and infections may offset its benefits by increasing non-relapse mortality (NRM) [3]. Randomized trials have demonstrated that pre-transplant anti-T-Lymphocyte globulin (ATLG) can reduce severe acute and chronic-graft-versus-host disease (a/cGVHD) [4–6]. While lower doses of ATLG may compromise its immunosuppressive effects, higher doses could offset its benefits by reducing antiviral efficacy and graft-versus-malignancy effects through the depletion of donor effector T-cells [7]. Despite the established use of ATLG in allo-SCT, no studies have compared ATLG dosing specifically in the context of MSD-PBSCT, and only two studies have compared different ATLG doses in MUD-PBSCT [8, 9]. In this study, we aim to compare transplant outcomes between 15 mg/kg versus 30 mg/kg ATLG as an in vivo T-cell depletion (TCD) strategy for patients undergoing MSD-PBSCT for hematological malignancies.

## Materials and methods

This retrospective study conducted at University Medical Center Hamburg-Eppendorf (UKE) with a primary goal to compare cGVHD between 15 mg/kg (ATLG-15) and 30 mg/kg (ATLG-30) ATLG in recipients of (HLA 10/10) MSD-PBSCT. Secondary outcomes included engraftment, aGVHD, NRM, cumulative incidence of relapse (CIR), progression free survival (PFS), overall survival (OS) and graftversushostdisease, relapse-free survival (GRFS).

## Ethics approval and consent to participate

This study was approved by the Ethics Committee of the University Medical Center Hamburg-Eppendorf (UKE) (reference number: 2022-100940-BO-ff). The study was performed in accordance with the Declaration of Helsinki. Informed consent was obtained from all participants.

Myeloablative conditioning (MAC) regimens were defined according to published working group definition [10]. ATLG (Grafalon®, Neovii, Switzerland) was given at a dose of 15 mg/kg or 30 mg/kg. ATLG (Grafalon®, Neovii, Switzerland) was administered with a test dose of 200 mg on day −4, and the remaining doses were fractionated between days −3 and −1. Posttransplant GVHD prophylaxis consisted of ciclosporine A from day −1. The ATLG dose was selected based on physician preference, with a trend toward using lower doses (15 mg/kg) in more recent years. To account for disease-related heterogeneity, the Disease Risk Stratification System (DRSS) was used as a covariate in all multivariate and propensity score models. For the analysis of NRM, the DRSS was regrouped to enable stable estimation in the competing risks model, as no NRM events occurred in the Standard-risk group. Specifically, Standard, Intermediate-1, and Intermediate-2 were combined into a Low-risk category, while High and Very High were grouped as High-risk. This regrouping was applied only for the NRM model. For all other endpoints, the original DRSS categories were retained [11]. Neutrophil engraftment was defined as the first 3 consecutive days with a measure of absolute neutrophil count >0.5 × 10^9^/L. Platelet engraftment was defined as the first 3 consecutive days with a platelet count > 20 × 10^9^/L without transfusion support. Acute GVHD was graded according to standard criteria [12]. Chronic GVHD was graded according to National Institute of Health (NIH) criteria routinely at every visit after transplantation [13].

All outcomes were measured from the time of allo-SCT. PFS was defined as the duration of survival without relapse or progression, with censoring for patients without these events at last follow-up. OS was defined as survival without death from any cause, while NRM was defined as death without evidence of relapse. For statistical analysis, Kaplan–Meier methods were employed to estimate probabilities for PFS and OS, with group differences assessed via the log-rank test. Cumulative incidence functions were used to estimate engraftment, CIR, NRM, aGVHD, and cGVHD in a competing risk framework. Specifically, CIR and NRM were treated as competing events, and death without the respective event was considered the competing risk for aGVHD, cGVHD, and engraftment. GRFS was defined as survival without disease relapse or progression and without Grade III-IV aGVHD or moderate to severe cGVHD.

For univariate analyses, continuous variables were categorized. Univariate comparisons were performed using the log-rank test and Gray’s test for cumulative incidences. Multivariate analysis (MVA) was conducted using a Cox proportional hazards model to calculate adjusted hazard ratios and 95% confidence intervals. A p value of less than 0.05 was considered statistically significant. MVA using Fine and Gray’s competing risks regression model was performed to identify independent prognostic factors for NRM, CIR, GVHD, and engraftment. Variables with a *p* value less than 0.1 or clinically relevant were included.

To ensure comparability between the ATLG-15 and ATLG-30 groups for confounding variables, we employed propensity score matching (PSM) using the MatchIt package in R. Due to the limited number of cases and potential issues with perfect separation, we excluded patients with an ECOG performance status of 2 and those who received total body irradiation (TBI) to create a more homogeneous study population. The final matching model included the following covariates: ECOG performance status, year of allogeneic SCT, patient age, patient CMV serology, conditioning regimen intensity (MAC vs. RIC), and a grouped version of the Disease Risk Stratification System (DRSS), where DRSS standard, intermediate 1 and intermediate 2 were combined and compared high and very high. Matching was performed using nearest neighbor matching with a 1:2 ratio and a caliper width of 0.2 to ensure stricter match quality. The balance of covariates between the matched groups was assessed using standardized mean differences (SMD) and visualized with love plots generated using the cobalt package. The resulting matched dataset was used for all subsequent outcome analyses.

To explore whether the observed associations between ATLG dose and outcomes were influenced by conditioning intensity, we performed stratified univariate analyses in a more homogeneous subgroup of the full cohort. Specifically, we identified patients with AML or MDS who received conditioning without TBI, and further stratified them by conditioning intensity (MAC vs. RIC). Within these subgroups, outcomes were compared between patients who received ATLG 15 mg/kg versus 30 mg/kg. Survival outcomes (OS, PFS, GRFS) were analyzed using Kaplan-Meier estimates and the log-rank test. Cumulative incidence analyses were performed for NRM, relapse, acute and chronic GVHD, using Gray’s test to account for competing risks. The goal was to isolate the effect of ATLG dose within clinically relevant and biologically homogeneous subpopulations. To address potential confounding from disease heterogeneity and variations in conditioning intensity, additional subgroup analyses were conducted. The cohort was first restricted to patients with AML or MDS to reduce diagnosis-related variability. Within this subgroup, we further stratified patients based on conditioning intensity (MAC vs. RIC) to enable comparison of ATLG dosing in more homogeneous clinical contexts. Univariate analyses were performed within each stratum to evaluate the association between ATLG dose (15 mg/kg vs. 30 mg/kg) and transplant outcomes, including OS, PFS, GRFS, NRM, relapse, and acute and chronic GVHD. These stratified analyses aimed to isolate the effect of ATLG dose under more uniform clinical conditions. All statistical analyses were conducted using R (version 3.0, R Development Core Team, Vienna, Austria; https://www.r-project.org/). The following R packages were used: survival, cmprsk, ggplot2, dplyr, survminer, mstate, tableone, matchit, cobalt, and forestplot.

## Results

### Patients and transplant characteristics

A total of 165 consecutive patients were included in the study. Seventy-one patients received ATLG-15, and 94 Patients received ATLG-30. The median year of transplant was 2020 (2018–2022) in the ATLG-15 group and 2018 (2011–2022) in the ATLG-30 group (*p* < 0.001). The median age at transplant was 57 years (range, 20–72) and 58 years (range, 20–71) in the ATLG-15 and ATLG-30 (*p* = 0.88) groups, respectively. ECOG performance status scores were higher in the ATLG-15 group (ECOG0 18%, ECOG1 72%, ECOG2 9%) compared to the ATLG-30 group (ECOG0 36%, ECOG1 64%) (*p* = 0.001). Sixty eight percent received MAC in the ATLG-15 group compared to 48% in the ATLG-30 group (*p* = 0.01). All patients, donors, and transplant characteristics are listed in Table 1.

**Table 1:** Patients Donors and Transplants characteristics.

ATLG Dose	15 mg/Kg	30 mg/Kg	*P*
	*N* (%)	*N* (%)	
Total Patients	71 (100)	94 (100)	
Patient age median (range)	57 (20–72)	58 (20–71)	0.88
≤58 Years	40 (56)	49 (52)	0.6
>58 Years	31 (44)	45 (48)	
Disease			**<0.001**
**Acute Leukemia**	**39 (55)**	**30 (32)**	
ALL	0 (0)	1 (1)	
AML	39 (55)	29 (31)	
**MDS/MPN**	**26 (37)**	**19 (20)**	
CML	1 (1)	3 (3)	
MDS	18 (25)	14 (15)	
MDS/MPN	7 (10)	2 (2)	
**Others**	**39 (55)**	**30 (32)**	
MM	0 (0)	4 (4)	
NHL	6 (9)	6 (6)	
Histiocytosis	0 (0)	1 (0.01)	
**PMF**	0 (0)	34 (36)	
DRSS			**<0.0001**
Standard	7 (10)	12 (13)	
Intermediate 1	25 (35)	18 (19)	
Intermediate 2	11 (16)	46 (50)	
High	16 (23)	4 (4)	
Very High	12 (17)	13 (14)	
Unknown	0 (0)	1 (0.1)	
ECOG			
0	13 (18)	34 (36)	**0.001**
1	51 (72)	60 (64)	
2	7 (9)	0 (0)	
KI at SCT median (range)	80 (50–100)	80 (80–100)	**0.001**
Donor Age median (range)	57 (28–70)	58 (18–79)	0.5
≤57 Years	41 (58)	47 (50)	0.32
>57 Years	30 (42)	47 (50)	
Recipient/Donor CMV Serology			
-/-	14 (20)	26 (28)	0.44
-/+	10 (14)	17 (18)	
+/+	31 (44)	31 (33)	
+/-	16 (23)	20 (21)	
Recipient-Donor sex			0.17
MM	20 (28)	32 (34)	
MF	21 (30)	25 (27)	
FF	11 (16)	23 (25)	
FM	19 (27)	14 (15)	
ABO incompatibility			
Isogroup	50 (70)	71 (76)	0.16
Minor	9 (13)	6 (6)	
Major	12 (17)	13 (14)	
Bidirectional	0 (0)	4 (4)	
Allo-SCT Year	2020 (2018–2022)	2018 (2011–2022)	**<0.001**
≤ 2019	35 (49)	83 (88)	**<0.001**
> 2019	36 (51)	11 (12)	
CD34x10^6^/kg median (range)	5.1 (2.5–11.7)	5.8 (1.9–11.2)	**0.02**
≤5.7 ×10^6^/kg	43 (61)	39 (43)	**0.025**
>5.7 ×10^6^/kg	28 (39)	52 (57)	
Conditioning intensity			
MAC	48 (68)	45 (48)	**0.011**
RIC	23 (32)	49 (52)	
TBI	9 (13)	5 (5)	0.09
2 Gy	0 (0)	1 (20)	
8 Gy	8 (89)	0 (0)	
9 Gy	0(0)	1 (20)	
12 Gy	1 (11)	3 (60)	
Conditioning Regimen			**<0.001**
Bu-Cy	0 (0)	6 (6)	
Bu-Flu	17 (24)	55 (59)	
Bu-Flu-other	0 (0)	2 (2)	
Bu-TT	27 (38)	1 (1)	
FLAMSA-BuFlu	4 (6)	11 (12)	
FLAMSA-other	0 (0)	1 (1)	
FLAMSA-Treo	1 (1)	0 (0)	
Flu-Mel	0 (0)	2 (2)	
LEAM	0 (0)	1 (1)	
other	0 (0)	2 (2)	
TBI-Cy	0 (0)	1 (1)	
TBI-Flu	9 (13)	1 (1)	
TBI-Vp	0 (0)	1 (1)	
TMI-BuCy	0 (0)	1 (1)	
Treo-Flu	11 (15)	9 (10)	
Treo-FluTT	1 (1)	0 (0)	
Treo-TT	1 (1)	0 (0)	
Immune suppression			**0.73**
CSA + MMF	66 (93)	90 (96)	
Tac+MMF	4 (6)	3 (3)	
other	1 (1)	1 (1)	
**Disease status at SCT**			**0.06**
**CR**	**31 (44)**	**24 (26)**	
CR1	28 (90)	22 (92)	
CR2	3 (10)	2 (8)	
**PR**	**4 (6)**	**12 (13)**	
**Progression**	**16 (23)**	**18 (19)**	
Relapse	4 (6)	3 (3)	
PD	2 (3)	4 (4)	
Primary refractory	9 (13)	10 (11)	
Rel Refractory	1 (1)	1 (1)	
**Untreated**	**20 (28)**	**39 (42)**	
**Unknown**	**0 (0)**	**1 (1)**	

**Red**: *p* < 0.05.

*ATLG* anti-T-lymphocyte globulin, *ALL* acute lymphoblastic leukemia, *AML* acute myeloid leukemia, *MDS* myelodysplastic syndrome, *MPN* myeloproliferative neoplasm, *CML* chronic myeloid leukemia, *MM* multiple myeloma, *NHL* non-Hodgkin’s lymphoma, *PMF* primary myelofibrosis, *ECOG* Eastern Cooperative Oncology Group Performance Status, *KI* Karnofsky Index (Performance Status), *SCT* stem cell transplant, *CMV* cytomegalovirus, *ABO* ABO Blood Group, *MAC* myeloablative conditioning, *RIC* Reduced-Intensity Conditioning, *TBI* Total Body Irradiation, *Bu* Busulfan, *Cy* Cyclophosphamide, *Flu* Fludarabine, *Mel* Melphalan, *Treo* Treosulfan, *TT* Thiotepa, *CSA* Ciclosporine, *MMF* Mycophenolate Mofetil, *Tac* Tacrolimus, *CR* Complete Remission, *CR1* First Complete Remission, *CR2* Second Complete Remission, *PR* Partial Remission, *PD* Progressive Disease, *Rel* Relapse, Rel Refractory: Refractory Relapse, *LEAM* Lomustine–Etoposide–Ara-C–Melphalan, *TMI* Total Marrow Irradiation, *Vp* Etoposide, *Allo-SCT* Allogeneic Stem Cell Transplant, *N* Number of patients, *%* Percentage, *p* *p* value.

#### Transplant outcomes

All univariate analysis for transplant outcomes are summarized in Table 2.

**Table 2:** Univariate analysis.

	cGVHD all Grade	cGVHD mod/sev	aGVHD G III-IV	aGVHD G II-IV	NRM	CIR	GRFS	PFS	OS
	*p*	*p*	*p*	*p*	*p*	*p*	*p*	*p*	*p*
	2 y [95%CI]	2 y [95%CI]	D100 [95%CI]	D100 [95%CI]	2 y [95%CI]	2 y [95%CI]	2 y [95%CI]	2 y [95%CI]	2 y [95%CI]
**ATLG Dose**	0.21	**0.045**	0.70	0.20	0.11	0.64	0.10	0.40	0.20
15 mg/Kg	73 [57–83]	43 [31–55]	13 [6–22]	34 [23–45]	13 [6–22]	25 [15–37]	24 [14–39]	60 [49–75]	72 (61–84)
30 mg/Kg	62 [50–72]	28 [19–39]	9 [4–16]	18 [11–27]	6 [2–13]	28 [19–39]	36 [27–49]	65 [55–76]	77 (68–87)
Patient Age	0.21	0.87	0.31	**0.09**	**0.02**	0.68	**0.02**	**0.04**	**0.01**
≤58 Years	74 [61–82]	36 [26–47]	8 [3–15]	19 [12–28]	3 [5–9]	26 [17–37]	36 [26–50]	71 [61–82]	82 [74–92]
>58 Years	57 [43–68]	33 [22–45]	14 [7–23]	32 [21–43]	16 [8–26]	28 [18–40]	24 [16–38]	53 [42–67]	66 [55–79]
**ECOG**	0.37	0.70	0.15	0.34	0.87	0.95	0.40	0.5	**0.05**
0	75 [57–86]	37 [22–52]	6 [1–16]	21 [11–34]	5 [1–16]	28 [15–43]	29 [18–49]	66 [53–83]	80 [69–94]
1	63 [52–73]	33 [24–43]	13 [8–20]	25 [18–34]	10 [5–17]	26 [18–37]	33 [24–45]	63 [54–74]	75 [66–85]
2	NR	NR	0	43 [7–76]	14 [1–50]	32 [3–70]	14 [2–88]	43 [18–100]	43 [18–1]
**Donor Age**	0.64	0.34	0.66	0.39	0.77	0.52	0.40	0.50	0.30
≤57 Years	64 [52–73]	30 [21–41]	11 [6–19]	30 [20–39]	9 [4–16]	25 [16–35]	36 [27–49]	65 [55–77]	77 [69–87]
>57 Years	69 [54–80]	42 [29–54]	10[4–18]	19 [11–29]	9 [4–18]	31 [19–43]	23 [14–39]	60 [49–74]	72 [61–84]
**Patient CMV serology**	0.63	0.93	0.52	**0.007**	0.29	**0.01**	0.40	**0.07**	0.20
Neg	65 [51–76]	35 [23–48]	6 [2–14]	12 [6–21]	5 [1–13]	37 [24–49]	25 [15–40]	57 [45–71]	67 [56–81]
Pos	66 [54–76]	35 [25–45]	14 [8–22]	33 [24–43]	12 [6–19]	21 [13–31]	36 [26–48]	67 [58–79]	80 [72–90]
**Donor CMV Serology**	0.89	0.71	0.91	0.10	0.92	0.94	0.40	0.90	0.80
Neg	64 [51–75]	37 [25–49]	12 [6–21]	23 [14–33]	10 [4–18]	24 [14–34]	32 [23–46]	66 [56–78]	71 [62–83]
Pos	67 [54–77]	33 [23–44]	9 [4–17]	27 [18–37]	8 [3–16]	31 [20–42]	31 [21–44]	60 [50–73]	78 [69–88]
**Patient Sex**	0.81	0.61	0.73	0.71	0.56	0.91	0.50	0.90	0.60
Male	67 [55–77]	33 [24–44]	11 [5–18]	24 [16–33]	8 [4–16]	29 [20–40]	59 [22–44]	61 [51–73]	73 [63–83]
Female	64 [50–75]	37 [25–50]	11 [5–19]	26 [16–37]	10 [4–19]	24 [14–35]	32 [22–48]	66 [55–79]	78 [68–90]
**Donor Sex**	0.43	0.90	0.62	**0.08**	**0.06**	0.98	0.50	0.30	0.20
Male	65 [51–76]	33 [22–43]	11 [5–19]	20 [12–29]	6 [2–13]	26 [17–37]	36 [26–49]	67 [57–79]	79 [69–90]
Female	67 [54–77]	36 [25–48]	10 [5–18]	30 [21–41]	12 [6–21]	28 [18–39]	28 [18–41]	60 [49–72]	71 [61–82]
**ABO Incompatibility**	0.31	0.10	0.77	0.20	0.91	0.53	0.50	0.30	0.60
Isogroup	63 [52–72]	33 [24–42]	10 [5–16]	23 [15–30]	9 [5–16]	28 [20–37]	32 [24–43]	62 [53–72]	73 [65–83]
Minor	75 [35–92]	57 [24–81]	15 [2–40]	46 [18–71]	8 [1–30]	23 [5–49]	19 [6–64]	69 [48–99]	69 [45–100]
Major	76 [49–90]	39 [19–59]	13 [3–29]	25 [10–44]	9 [1–25]	19 [5–39]	31 [16–61]	72 [55–94]	82 [68–99]
Bidirectional	NR	0	0	25 [1–71]	NR	NR	NR	NR	NR
**Allo-SCT Year**	0.82	0.89	0.12	0.43	0.57	0.3	0.8	0.7	0.6
≤ 2019	35 [26–44]	65 [54–73]	8 [4–14]	24 [16–32]	8 [4–14]	29 [21–38]	32 [24–43]	63 [54–73]	74 [66–83]
> 2019	35 [19–50]	74 [36–92]	16 [7–29]	28 [16–42]	11 [3–24]	25 [8–45]	25 [10–61]	62 [45–85]	77 [63–95]
**CD34 Dose**	0.83	0.29	0.23	0.40	0.89	0.31	0.10	0.40	0.30
≤5.7 ×10^6^/kg	68 [54–78]	39 [28–51]	13 [6–21]	29 [20–39]	10 [4–18]	33 [22–45]	22 [13–36]	57 [46–71]	71 [61–83]
>5.7 ×10^6^/kg	63 [50–74]	31 [20–42]	8 [3–15]	20 [12–29]	8 [3–16]	22 [13–32]	40 [30–53]	68 [58–80]	78 [69–89]
**Conditioning intensity**	0.37	0.90	**0.0006**	0.18	0.38	0.91	**0.02**	0.40	0.99
MAC	71 [58–80]	34 [24–44]	4 [1–10]	21 [13–30]	6 [2–12]	28 [18–39]	37 [27–50]	66 [56–78]	77 [68–87]
RIC	60 [46–72]	35 [24–48]	19 [10–29]	30 [20–41]	13 [6–23]	26 [16–38]	22 [14–38]	59 [48–73]	72 [61–85]
**TBI**	0.59	0.71	0.52	0.47	0.57	**0.005**	0.10	**0.001**	**0.0002**
TBI	67 [58–75]	35 [27–43]	11 [7–17]	25 [19–33]	8 [4–14]	24 [17–31]	34 [26–43]	67 [60–76]	78 [71–86]
no TBI	54 [22–77]	31 [9–57]	7 [1–28]	21 [5–46]	16 [2–41]	64 [27–87]	10 [2–58]	20 [6–65]	45 [24–84]
**Disease status at SCT**	0.12	0.28	0.34	0.80	0.42	**0.004**	0.20	**0.0003**	**0.0004**
CR	73 [56–84]	38 [24–52]	6 [1–14]	25 [14–37]	4 [1–12]	22	39 [27–58]	71 [59–86]	78 [67–91]
PR	58 [27–80]	36 [12–61]	6 [1–25]	31 [11–54]	21 [5–45]	21 [11–36]	27 [12–63]	58 [38–91]	73 [53–99]
PD	45 [26–61]	19 [7–34]	9 [2–22]	19 [7–34]	13 [4–27]	55 [5–45]	20 [9–41]	32 [19–55]	52 [37–73]
Untreated	73 [57–84]	43 [28–56]	17 [9–28]	27 [17–39]	8 [3–19]	17 [35–71]	31 [19–48]	75 [64–88]	87 [77–97]
**CR vs not CR**	0.76	0.42	**0.07**	0.87	0.28	0.49	0.10	0.30	0.50
CR	73 [56–84]	38 [24–52]	6 [1–14]	25 [14–37]	4 [1–12]	23 [11–36]	39 [27–58]	71 [59–86]	78 [67–91]
not CR	62 [51–70]	33 [24–42]	13 [7–20]	25 [17–34]	11 [6–19]	29 [21–39]	27 [19–38]	59 [50–70]	73 [65–83]
**DRSSS**					0.525	**<0.001**	**0.005**	**<0.001**	**0.005**
Standard					0 [NA]	16 [4–36]	39 [21–76]	81 [64–100]	89 [75–100]
Intermediate 1					9 [3–20]	14 [6–26]	40 [26–61]	70 [56–88]	79 [65–95]
Intermediate 2					11 [4–20]	21 [12–32]	30 [19–47]	64 [52–79]	75 [63–88]
High					5 [0–21]	15 [4–34]	43 [26–72]	72 [54–97]	84 [69–100]
Very High					8 [1–23]	60 [37–77]	5 [1–33]	28 [14–55]	52 [35–78]

*cGVHD all Grade* Chronic Graft-versus-Host Disease (all grades), *cGVHD mod/sev* Chronic Graft-versus-Host Disease (moderate/severe), *aGVHD G III–IV* Acute Graft-versus-Host Disease (Grade III–IV), *aGVHD G II–IV* Acute Graft-versus-Host Disease (Grade II–IV), *NRM* Non-Relapse Mortality, *CIR* Cumulative Incidence of Relapse, *GRFS* Graft-versus-Host Disease–Free, Relapse-Free Survival, *PFS* Progression-Free Survival, *OS* Overall Survival, *2* *y* 2-year outcome, *D100* Day 100 outcome, [95% CI]: 95% Confidence Interval, *ATLG* Anti-T-Lymphocyte Globulin, *ECOG* Eastern Cooperative Oncology Group performance status, *CMV* cytomegalovirus, *ABO* ABO blood group, *Allo-SCT* Allogeneic Stem Cell Transplant, *CD34 Dose* Infused CD34⁺ cell dose (×10^6/kg), *MAC* Myeloablative Conditioning, *RIC* Reduced-Intensity Conditioning, *TBI* Total Body Irradiation, *CR* Complete Remission, *PR* Partial Remission, *PD* Progressive Disease, *NR* Not Reached (for survival estimates), ***p***: *p* value.

#### Engraftment

One patient died prior to engraftment and three patients had primary graft failure in the ATLG-15 group, all remaining patients successfully engrafted. The ATLG-15 cohort showed an earlier leukocyte engraftment (median 11 days, range 8–19, *p* = 0.004) and earlier platelet engraftment (median 12 days, range 8–107, *p* = 0.0002) compared to the other group’s medians of 12 days (range 8–16) and 15 days (range 3–249), respectively.

#### GVHD

**Fig. 1:**
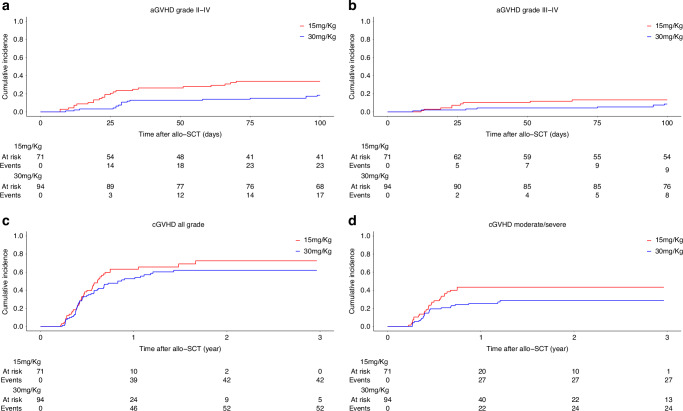
Graft versus host disease. ATLG 15 mg/Kg vs 30 mg/Kg. **a** aGVHD grade II-IV. **b** aGVHD grade III-IV. **c** cGVHD all grade. **d** cGVHD moderate/Severe.

The cumulative incidence of aGVHD grade II-IV at day 100 was comparable between the two groups (ATLG-15: 34% vs ATLG-30: 18%, *p* = 0.2) (Fig. 1a). Only patients’ CMV serology significantly affected aGVHD II-IV, with a cumulative incidence of 12% in patients with negative serology compared to 33% in those with positive serology on univariate analysis (*p* = 0.007). This difference persisted on MVA (HR: 2.40 [95% CI: 1.30–4.43], *p* = 0.005). Additionally, there was a trend for increased risk of aGVHD II-IV in patients transplanted from female donors versus male donors (HR: 1.75 [95% CI: 0.96, 3.18], *p* = 0.068).

The cumulative incidence of aGVHD Grade III-IV at day 100 was comparable between the ATLG-15 (13%) and the ATLG-30 (9%) groups (*p* = 0.7) (Fig. 1b). Conversely, only conditioning intensity significantly affected aGVHD Grade III-IV, with a cumulative incidence of 4% in patients who received MAC and 19% in those who received RIC (*p* = 0.0006). This difference persisted on MVA (HR: 5.89 [95% CI: 1.71–20.26], *p* = 0.0049).

We observed no differences in the cumulative incidence of all grade cGVHD between the two groups, with a cumulative incidence at 2 years of 73% versus 62% in the ATLG-15 and ATLG-30 groups, respectively (*p* = 0.21) (Fig. 1c). On MVA, none of the factors affected all grade cGVHD.

Patients in the ATLG-15 group had a significantly higher cumulative incidence of moderate/severe cGVHD compared to patients in the ATLG-30 group (ATLG-15: 43% vs ATLG-30: 28%, *p* = 0.045) (Fig. 1d). No other factor significantly affected cGVHD. This difference persisted on MVA (HR: 0.450 [95% CI: 0.214, 0.946], *p* = 0.035). MVA for GVHD are summarized in Table 3.

**Table 3:** Multivariate analysis graft versus host disease, non relapse mortality and cumulative incidence of relapse.

	NRM
	HR [95% CI] *p* value
Patient age per 1 yr increase	1.048 [0.976, 1.120] 0.20
ATLG-30 vs ATLG-15	0.398 [0.122, 1.300] 0.13
DRSS High-risk vs Low-risk	0.631 [0.176, 2.250] 0.48
	CIR
	HR [95% CI] *p* value
Patient Age (per 1 yr increase)	1.021 [0.994, 1.050] 0.12
ATLG-30 vs ATLG-15	1.314 [0.661, 2.610] 0.44
Donor Sex: Female vs Male	0.854 [0.450, 1.620] 0.63
DRSS: Intermediate 1 vs Standard	0.947 [0.287, 3.120] 0.93
DRSS: Intermediate 2 vs Standard	1.133 [0.372, 3.450] 0.83
DRSS: High vs Standard	0.891 [0.193, 4.120] 0.88
**DRSS: Very High vs Standard**	**3.370 [1.102, 10.310] 0.03**
**TBI: TBI vs no TBI**	**2.507 [1.040, 6.040] 0.04**

	aGVHD24	aGVHD34
	HR [95% CI] *p* value	HR [95% CI] *p* value
Patient age per 1 yr increase	1.015 [0.987, 1.04] 0.300	1.023 [0.975, 1.07] 0.3600
Year of Transplant per 1 yr increase	1.018 [0.837, 1.24] 0.860	0.987 [0.698, 1.40] 0.9400
ATLG-30 vs ATLG-15	0.708 [0.340, 1.47] 0.360	0.623 [0.216, 1.79] 0.3800
**Patient CMV serology pos vs neg**	**2.403 [1.303, 4.43] 0.005**	1.608 [0.652, 3.97] 0.3000
**Donor sex Female vs Male**	**1.747 [0.959, 3.18] 0.068**	1.388 [0.562, 3.43] 0.4800
RIC vs MAC	1.603 [0.859, 2.99] 0.140	**5.893 [1.714, 20.26] 0.0049**
	cGVHD all	cGVHD moderate/Severe
	HR [95% CI] p value	HR [95% CI] p value
Patient age per 1 yr increase	0.993 [0.976, 1.01] 0.48	1.004 [0.977, 1.031] 0.780
Year of Transplant per 1 yr increase	1.002 [0.880, 1.14] 0.98	0.922 [0.792, 1.073] 0.290
ATLG-30 vs ATLG-15	0.810 [0.463, 1.41] 0.46	**0.450 [0.214, 0.946] 0.035**
RIC vs MAC	0.814 [0.474, 1.40] 0.46	1.185 [0.656, 2.141] 0.570
No TBI vs TBI	0.760 [0.334, 1.73] 0.51	
CR vs not CR	1.106 [0.649, 1.89] 0.71	
CD34 Dose	0.935 [0.851, 1.03] 0.15	

*CIR* Cumulative Incidence of Relapse, *NRM* Non-Relapse Mortality, *HR* Hazard Ratio, *CI* Confidence Interval, *CR* Complete Remission, *TBI* Total Body Irradiation, *aGVHD24* Acute Graft-versus-Host Disease (Grades 2–4), *aGVHD34* Acute Graft-versus-Host Disease (Grades 3–4), *cGVHD* Chronic Graft-versus-Host Disease, *ATLG* Anti-T-Lymphocyte Globulin, *RIC* Reduced-Intensity Conditioning, *MAC* Myeloablative Conditioning, *CMV* Cytomegalovirus, *CD34 Dose* Infused CD34⁺ Cell Dose (×10⁶ cells/kg), *DRSS* Disease Risk Stratification System (Standard, Intermediate 1/2, High, Very High; grouped into Low-risk vs High-risk for NRM analysis).

Red: *p* < 0.05, Bold: *p* < 0.1.

#### OS and PFS

**Fig. 2:**
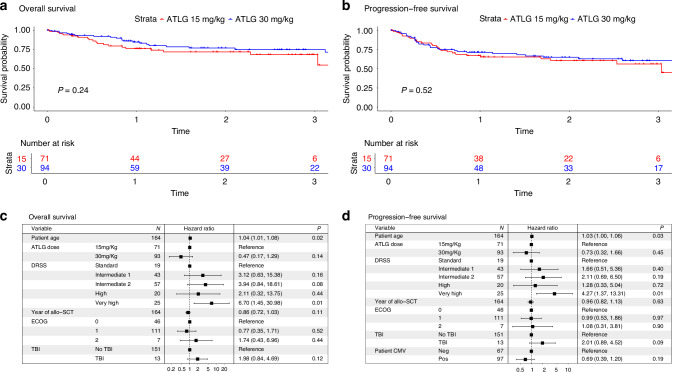
Overall survival and progression free survival ATLG 15 mg/Kg vs 30 mg/Kg. **a** OS. **b** PFS. **c**Multivariate analysis OS. **d** Multivariate analysis PFS.

The estimated 2-year OS was 72% for patients in the ATLG-15 group and 77% in the ATLG-30 group (*p* = 0.2) (Fig. 2a). The estimated 2-year PFS was 60% for patients in the ATLG-15 group and 65% in the ATLG-30 group (*p* = 0.4) (Fig. 2b). On univariate analysis older patients, TBI, higher DRSS and progressive disease at transplant were associated with lower OS and PFS. There was a trend for lower OS for patients with higher ECOG performance status and lower PFS for patients with negative CMV serology. No other factor affected OS or PFS on univariate analysis. In MVA, older age (HR 1.04 [95%CI: 1.01–1.08], *p* = 0.02) and Very High DRSS risk vs. Standard (HR 6.70 [95%CI: 1.45–30.98], *p* = 0.01) were significantly associated with inferior OS. (Fig. 2c) For PFS, older age (HR 1.03 [95%CI: 1.00–1.06], *p* = 0.03) and Very High DRSS risk vs. Standard (HR 4.27 [95%CI: 1.37–13.31], *p* = 0.01) were also significantly associated with worse outcomes. (Fig. 2d)

#### NRM and CIR

**Fig. 3:**
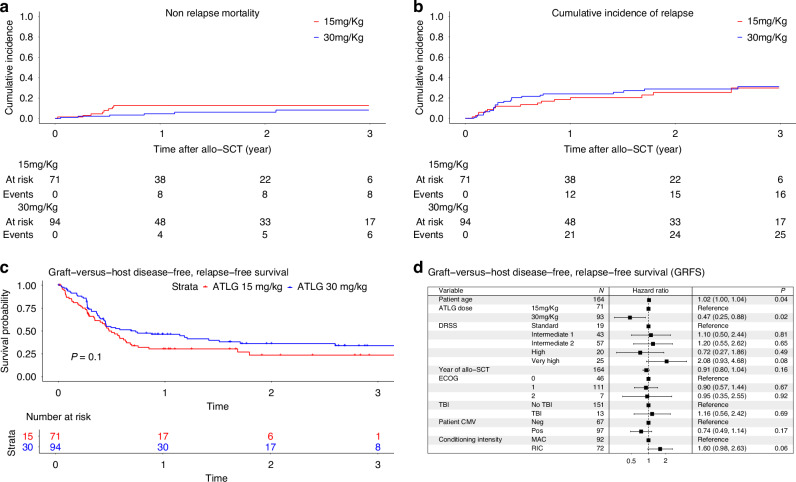
Non relapse mortality, cumulative incidence of relapse, graft versus host disease relapse free survival ATLG 15 mg/Kg vs 30 mg/Kg. **a** Non relapse mortality. **b** Cumulative incidence of relapse. **c** GRFS. **d** Multivariate analysis GRFS.

The 2-year cumulative incidence of NRM was comparable between the two groups, with 13% in the ATLG-15 group and 6% in the ATLG-30 group (*p* = 0.11) (Fig. 3a). Only patients’ age negatively impacted NRM on univariate analysis. On MVA none of the factors impacted NRM (Table 3).

The CIR at 2 years was similar between the ATLG-15 and ATLG-30 groups, with rates of 25% and 28%, respectively (*p* = 0.64). (Fig. 3b) However, we observed a higher CIR in patients with negative CMV serology compared to those with positive CMV serology (at 2 years: CMV neg 37% vs CMV pos 21%, *p* = 0.01). Patients who received total body irradiation (TBI) had a significantly higher CIR at 2 years compared to those who did not receive TBI (64% vs 24%; *p* = 0.005). Additionally, patients transplanted with progressive disease (PD) exhibited a higher CIR at 2 years (PD: 55%) compared to those in complete remission (CR: 22%), partial remission (PR: 21%), or untreated (17%) (*p* = 0.004). The cumulative incidence of relapse was highest in the Very High DRSS group at 60 [95%CI: 37–77], compared to 16 [95%CI: 4–36] in Standard, 14 [95%CI: 6–26] in Intermediate 1, 21 [95%CI: 12–32] in Intermediate 2, and 15 [95%CI: 4–34] in the High-risk group (*p* < 0.001). No other factors significantly impacted CIR in the univariate analysis. In MVA, Very High DRSS risk vs. Standard (HR 3.37 [95%CI: 1.10–10.31], *p* = 0.03) and TBI vs. no TBI (HR 2.51 [95%CI: 1.04–6.04], *p* = 0.04) were significantly associated with increased relapse risk.

#### GRFS

The GRFS at 2 years was comparable between the ATLG-15 and ATLG-30 groups, with rates of 24% and 36%, respectively (*p* = 0.1) (Fig. 3c). On univariate analysis, patient age (at 2 years: age ≤58 years 36% vs > 58 years 24%, *p* = 0.02) and RIC (at 2 years: MAC 37% vs RIC 22%, *p* = 0.02) negatively impacted GRFS. GRFS was markedly lower in the very high DRSS group at 2 yrs 5% [95%CI: 1–33], compared to 39% [95%CI: 21–76] in Standard, 40% [95%CI: 26–61] in Intermediate 1, 30% [95%CI: 19–47] in Intermediate 2, and 43 [95%CI: 26–72] in the High-risk group (*p* = 0.005).

In MVA, older age (HR 1.02 [95%CI: 1.00–1.04], *p* = 0.04) was significantly associated with inferior GRFS, while ATLG-30 vs. ATLG-15 was associated with improved GRFS (HR 0.47 [95%CI: 0.25–0.88], *p* = 0.02). Additionally, there was a trend toward inferior GRFS in patients receiving RIC compared to MAC (HR 1.60 [95%CI: 0.98–2.63], *p* = 0.06), and in those with Very High DRSS risk vs. Standard (HR 2.08 [95%CI: 0.93–4.68], *p* = 0.08). (Fig. 3d)

#### Propensity score matching

Propensity score matching yielded 19 matched cases and 31 matched controls. Covariate balance improved substantially after matching, with standardized mean differences for most variables falling well below 0.2. The variance ratio for the propensity scores was close to 1 (1.16), indicating strong balance between groups. Visual assessment using a Love plot and a density plot confirmed the reduction in imbalance across covariates (Supplementary Fig. 1A, B). In competing risk analysis, the 2-year cumulative incidence of non-relapse mortality was **30% in the ATLG-15 group** and **0% in the ATLG-30 group**. Due to the absence of NRM events in the ATLG-30 group, the sub distribution hazard could not be reliably estimated using Fine-Gray regression. No significant differences were observed in OS, PFS, CIR GRFS, aGVHD Grade II-IV, aGVHD III-IV, cGVHD all grade and moderate/severe. All results are summarized in Supplementary Table 1

#### Subgroup analysis of AML/MDS Patients who did not receive TBI by conditioning intensity

Among AML/MDS patients who did not receive TBI (*n* = 108), 61 received ATLG 15 mg/kg and 47 received ATLG 30 mg/kg. In the MAC subgroup (*n* = 69), 38 received 15 mg/kg and 31 received 30 mg/kg. In the RIC subgroup (*n* = 39), 23 received 15 mg/kg and 16 received 30 mg/kg.

In the overall AML/MDS no-TBI population, the incidence of moderate-to-severe cGVHD was significantly higher with ATLG 15 mg/kg compared to 30 mg/kg (38% vs. 19%, *p* = 0.039). In the MAC subgroup, moderate-to-severe cGVHD occurred more frequently with ATLG 15 mg/kg (39% vs. 19%, *p* = 0.079). In the RIC subgroup, no statistically significant differences were observed between ATLG 15 mg/kg and 30 mg/kg for any outcomes.

All results are summarized in Supplementary Table 2.

## Discussion

Although ATLG is recommended for GVHD prevention in allo-SCT [14], data on optimal dosing of ATLG in the setting of MSD-PBSCT is still lacking. A consensus recommendation by an international expert panel recommends the use of 30 mg/kg ATLG in MSD and 60 mg/KG in MUD allo-SCT [15].

This is the first study comparing ATLG doses in the MSD-PBSCT setting. Our findings demonstrate that the ATLG-30 was associated with a reduction in moderate to severe cGVHD and improved GRFS. Engraftment was faster in the ATLG-15 group; however, no significant differences were observed between the groups for aGVHD, CIR, NRM, PFS or OS. These results suggest that the ATLG-30 may provide a preferable balance between GVHD prevention and relapse risk without compromising key transplant outcomes.

Our results align with previous evidence demonstrating that pre-transplant ATLG can effectively reduce severe acute and chronic GVHD in allo-SCT [4–6]. Notably, although the overall incidence of aGVHD in our cohorts did not differ significantly, we observed a clear benefit in terms of lower moderate/severe cGVHD with ATLG-30. This is in line with prior work suggesting that sufficiently dosed ATLG might confer more pronounced immunomodulatory effects on donor T cells, thereby reducing the risk of severe cGVHD [7].

While multiple studies have reported the benefits of ATLG in MUD-PBSCT, only two have specifically compared different doses in that setting [8, 9]. In our previous study comparing 30 mg/kg (ATLG-30) vs 60 mg/kg (ATLG-60) ATLG in MUD-PBSCT, we reported an earlier engraftment in patients receiving ATLG-30, with no differences in other transplant outcomes [9]. Our current findings in MSD-PBSCT mirror this pattern, as we observed earlier engraftment in the ATLG-15 group. Another study published from our center compared ATLG-30 with ATLG-60 in the MUD-allo-SCT setting between 1997 and 2005, reporting a higher NRM in the ATLG-60 group with no differences in other outcomes [16]. In our study, NRM was comparable between the groups, which may be explained by improved HLA matching and supportive care over the years. Moreover, our data in MSD-PBSCT suggest that a 30 mg/kg dose achieves a favorable GVHD profile without significantly increasing CIR, indicating that donor type may play a role in modulating the impact of ATLG dosing.

A retrospective study from 2003 comparing two doses of ATLG (<60 mg/Kg vs 60 mg/Kg) in CML patients undergoing MUD allo-SCT reported improved OS and DFS in patients receiving ≥60 mg/Kg ATLG, attributing these differences to a higher incidence of severe aGVHD in the lower-dose group [17]. However, in that study, patients in the <60 mg/Kg arm received non-uniform dosing, with 58% receiving 20 mg/Kg and 36% receiving 40 mg/Kg, while our study in the MSD-PBSCT setting compared uniform doses of 15 mg/Kg and 30 mg/Kg. Moreover, differences in donor types and advancements such as improved HLA matching and supportive care over the years further complicate direct comparisons. These factors may explain why our results did not demonstrate significant differences in aGVHD or survival outcomes between the two dosing regimens.

Several studies have examined different doses of anti-thymocyte globulin (Thymoglobulin, ATG) in various transplant settings, highlighting the delicate balance between GVHD prevention and infection risks [18–21]. Butera et al. retrospectively compared two Thymoglobulin doses (5 mg/kg vs. 6.5 mg/kg) in adults undergoing MUD allo-SCT and found no significant differences in the long term transplant outcomes [18]. Bacigalupo et al. reported that a higher Thymoglobulin dose (15 mg/kg vs. 7.5 mg/kg) in MUD allo-SCT reduced acute GVHD (37% vs. 69%) [19]. Importantly, unlike ATLG, Thymoglobulin may also contain antibodies targeting thymus-specific cells, thus further impairing thymic T-cell regeneration [22]. Additional work in haplo-identical and cord blood transplant settings observed increased infection rates with higher ATLG doses [20, 21]. To refine dosing, it has been proposed that both body weight and absolute lymphocyte count (ALC) be considered, an approach that has effectively reduced GVHD and infection/relapse rates in two studies [23, 24]. A post hoc analysis of a randomized trial confirmed worse survival outcomes for patients with lower ALC on the first infusion day [25]. In our study, we did not evaluate the impact of ALC on outcomes.

Taken together, these findings underscore that optimal ATLG dosing should not only minimize GVHD but also preserve anti-leukemic immunity and maintain stable engraftment. Our observation of improved GRFS in the ATLG-30 group parallels results from other investigations highlighting the potential of higher ATLG doses to mitigate long-term complications [4–6, 16]. Future prospective trials focusing on patient-reported outcomes and immunologic monitoring could further elucidate the best dose to balance GVHD control and relapse, particularly in the MSD-PBSCT setting.

This is, to our knowledge, the first study comparing uniform ATLG doses (15 vs. 30 mg/kg) specifically in the MSD-PBSCT setting. Although retrospective and single-center in design, we sought to mitigate selection bias by employing propensity score matching, conducting a multivariate analysis and subgroup analyses in more homogeneous patient populations and conditioning regimens, thereby improving comparability and accounting for potential confounders. Nonetheless, the relatively low number of matched pairs remaining after propensity score matching substantially limits statistical power and increases the risk of overfitting or type II errors. These findings warrant validation in larger, multi-center randomized prospective trials. Going forward, efforts to refine ATLG dosing strategies should incorporate carefully stratified patient populations and could benefit from the inclusion of patient-reported outcomes to enhance our understanding of how different dosing regimens influence both clinical efficacy and quality of life.

Notably, our finding that TBI was associated with an increased risk of relapse contrasts with previous EBMT studies in AML and ALL, which reported a protective effect of TBI against relapse. In our analysis, this association remained significant even after adjustment for DRSS in the multivariate model. A likely explanation lies in the composition of our cohort: patients who received TBI predominantly had lymphoid malignancies, many of which were in advanced or refractory stages, or refractory AML. These disease contexts carry a high inherent relapse risk, which may have outweighed any potential antileukemic effect of TBI. Thus, the observed association likely reflects adverse disease biology and treatment resistance rather than a direct causal effect of TBI, and should be interpreted with caution. A limitation is potential confounding from evolving supportive care practices during 2011–2022, including letermovir introduction and enhanced antifungal strategies, with registry data showing 20-30% NRM reductions over similar periods [26–28]. While our single-institution standardized protocols minimize bias, incremental practice improvements may have contributed to the observed outcomes.

Contemporary transplant practice is evolving with recent evidence challenging traditional donor selection paradigms, particularly regarding the preference for older matched sibling donors over younger matched unrelated donors in elderly patients [29, 30].Additionally, post-transplant cyclophosphamide (PTCy) is rapidly expanding from haploidentical to all donor types, demonstrating the ability to eliminate HLA matching disparities and improve outcomes compared to traditional GVHD prophylaxis [31, 32]. These developments highlight the dynamic nature of transplant practice and the need for continued optimization of donor selection and GVHD prevention strategies alongside established approaches such as ATLG.

In conclusion, our study suggests that a 30 mg/kg ATLG dose in MSD-PBSCT can reduce moderate to severe cGVHD and potentially improve GRFS without significantly increasing relapse or NRM. Larger, multicenter trials are needed to confirm these observations and refine ATLG dosing in the MSD-PBSCT setting.

## Supplementary information

SUpplementary Material

## Data Availability

The analyzed data are available from the corresponding author on reasonable request.
