# Supplementary material for: Impact of anti-T-lymphocyte globulin dosing on graft versus host disease in matched sibling peripheral blood stem cell transplantation
Source: Bone Marrow Transplant. 2026 Jan 21;61(4):426–36. doi: 10.1038/s41409-025-02761-5 (PMC13056521; doi:10.1038/s41409-025-02761-5)

Supplementary Table 1, Transplant outcomes after Propensity Mask Score Matching

Outcome	Comparison	Estimate [95% CI], p
OS	ATLG-30 vs. 15	0.27 [0.03-2.24], 0.22
PFS	ATLG-30 vs. 15	0.67 [0.18-2.53], 0.55
GRFS	ATLG-30 vs. 15	0.58 [0.24-1.39], 0.22
REL	ATLG-30 vs. 15	3.25 [0.59-17.9], 0.18
aGVHD Grade II-IV	ATLG-30 vs. 15	0.42 [0.19-1.27], 0.14
aGVHD Grade III-IV	ATLG-30 vs. 15	0.25 [0.03-1.95], 0.19
cGVHD all grade	ATLG-30 vs. 15	0.66 [0.28-1.58], 0.34
cGVHD moderate/Severe	ATLG-30 vs. 15	0.14 [0.02-1.44], 0.06

Legend: ATLG: Anti-T-Lymphocyte Globulin, ATLG-30: 30 mg/kg ATLG, ATLG-15: 15 mg/kg ATLG, OS: Overall Survival, PFS: Progression-Free Survival, GRFS: Graft-versus-Host Disease–Free, Relapse-Free Survival, REL: Relapse, aGVHD: Acute Graft-versus-Host Disease, cGVHD: Chronic Graft-versus-Host Disease, HR: Hazard Ratio, CI: Confidence Interval

Supplementary Table 2: Subgroup analysis MDS/AML without Total Body irradiation.

ALL Population MDS/AML NO TBI		
**OS**		
**ATLG Dose**	**2y Estimate [95% CI]**	**p-value**
15 mg/kg	80 [95% CI: 70–91]	0.5
30 mg/kg	71 [95% CI: 58–86]	
**PFS**		
**ATLG Dose**	**2y Estimate [95% CI]**	**p-value**
15 mg/kg	80 [95% CI: 70–91]	0.5
30 mg/kg	71 [95% CI: 58–86]	
**GRFS**		
**ATLG Dose**	**2y Estimate [95% CI]**	**p-value**
15 mg/kg	28 [95% CI: 18–45]	0.2
30 mg/kg	42 [95% CI: 29–60]	
**NRM**		
**ATLG Dose**	**2y Estimate [95% CI]**	**p-value**
15 mg/kg	10 [95% CI: 4–19]	0.272
30 mg/kg	4 [95% CI: 1–13]	
**Relapse**		
**ATLG Dose**	**2y Estimate [95% CI]**	**p-value**
15 mg/kg	16 [95% CI: 8–27]	0.091
30 mg/kg	30 [95% CI: 17–43]	
**Relapse**		
**ATLG Dose**	**2y Estimate [95% CI]**	**p-value**
15 mg/kg	16 [95% CI: 8–27]	**0.091**
30 mg/kg	30 [95% CI: 17–43]	
**aGVHD Grade II-IV**		
**ATLG Dose**	**D100 Estimate [95% CI]**	**p-value**
15 mg/kg	34 [95% CI: 23–46]	0.111
30 mg/kg	15 [95% CI: 6–27]	
**aGVHD Grade III-IV**		
**ATLG Dose**	**D100 Estimate [95% CI]**	**p-value**
15 mg/kg	13 [95% CI: 6–23]	0.253
30 mg/kg	6 [95% CI: 2–16]	
**cGVHD all Grade**		
**ATLG Dose**	**2y Estimate [95% CI]**	**p-value**
15 mg/kg	59 [95% CI: 45–70]	0.139
30 mg/kg	47 [95% CI: 32–60]	
**cGVHD moderate/Severe**		
**ATLG Dose**	**2y Estimate [95% CI]**	**p-value**
15 mg/kg	38 [95% CI: 26–50]	0.039
30 mg/kg	19 [95% CI: 9–32]	

MAC MDS/AML No TBI		
**OS**		
**ATLG Dose**	**2y Estimate [95% CI]**	**p-value**
15 mg/kg	82 [95% CI: 70–96]	0.9

30 mg/kg	80 [95% CI: 66–96]	
PFS		
**ATLG Dose**	**2y Estimate [95% CI]**	**p-value**
15 mg/kg	70 [95% CI: 55–90]	0.9
30 mg/kg	76 [95% CI: 61–93]	
**GRFS**		
**ATLG Dose**	**2y Estimate [95% CI]**	**Column1**
15 mg/kg	30 [95% CI: 16–56]	0.31
30 mg/kg	54 [95% CI: 39–77]	
NRM		
**ATLG Dose**	**2y Estimate [95% CI]**	**p-value**
15 mg/kg	5 [95% CI: 1–16]	0.199
30 mg/kg	0 [CI not estimable]	
**Relapse**		
**ATLG Dose**	**2y Estimate [95% CI]**	**p-value**
15 mg/kg	18 [95% CI: 8–32]	0.459
30 mg/kg	23 [95% CI: 10–39]	
**aGVHD 24**		
**ATLG Dose**	**Estimate at Day 100 [95% CI]**	**p-value**
15 mg/kg	29 [95% CI: 15–44]	0.35
30 mg/kg	13 [95% CI: 4–27]	
**aGVHD 34**		
**ATLG Dose**	**Estimate at Day 100 [95% CI]**	**p-value**
15 mg/kg	5 [95% CI: 1–16]	0.695
30 mg/kg	3 [95% CI: 0–14]	
**cGVHD all Grade**		
**ATLG Dose**	**2y Estimate [95% CI]**	**p-value**
30 mg/kg	52 [95% CI: 32–68]	0.148
15 mg/kg	-	
**cGVHD moderate/Severe**		
**ATLG Dose**	**2y Estimate [95% CI]**	**p-value**
15 mg/kg	39 [95% CI: 24–55]	**0.079**
30 mg/kg	19 [95% CI: 8–35]	

**AML MDS No TBI RIC**		
**OS**		
**ATLG Dose**	**2y OS Estimate [95% CI]**	**p-value**
15 mg/kg	76 [95% CI: 60–97]	0.3
30 mg/kg	50 [95% CI: 28–90]	
**PFS**		
**ATLG Dose**	**2y DFS Estimate [95% CI]**	**p-value**
15 mg/kg	62 [95% CI: 45–87]	0.2
30 mg/kg	37 [95% CI: 19–75]	
**GRFS**		
**ATLG Dose**	**2y GRFS Estimate [95% CI]**	**p-value**
15 mg/kg	25 [95% CI: 12–52]	0.9
30 mg/kg	18 [95% CI: 6–57]	
**NRM**		
**ATLG Dose**	**2y Estimate [95% CI]**	**p-value**

15 mg/kg	17 [95% CI: 5–36]	0.668
30 mg/kg	13 [95% CI: 2–35]	
**RElapse**		
15 mg/kg	13 [95% CI: 3–30]	**0.083**
30 mg/kg	44 [95% CI: 19–67]	
**aGVHD II–IV**		
**ATLG Dose**	**Estimate at Day 100 [95% CI]**	**p-value**
15 mg/kg	43 [95% CI: 23–63]	0.184
30 mg/kg	19 [95% CI: 4–41]	
**aGVHD III–IV**		
**ATLG Dose**	**Estimate at Day 100 [95% CI]**	**p-value**
15 mg/kg	26 [95% CI: 10–45]	0.293
30 mg/kg	13 [95% CI: 2–34]	
**cGVHD all Grade**		
**ATLG Dose**	**Estimate at 2 Years [95% CI]**	**p-value**
15 mg/kg	48 [95% CI: 26–67]	0.527
30 mg/kg	38 [95% CI: 14–61]	
**Moderate–Severe cGVHD**		
**ATLG Dose**	**Estimate at 2 Years [95% CI]**	**p-value**
15 mg/kg	35 [95% CI: 16–54]	0.276
30 mg/kg	19 [95% CI: 4–41]	

ATLG: Anti-T-Lymphocyte Globulin, ATLG-30: 30 mg/kg ATLG, ATLG-15: 15 mg/kg ATLG,
OS: Overall Survival, PFS: Progression-Free Survival, GRFS: Graft-versus-Host Disease–Free, Relapse-Free Survival, NRM: Non-Relapse Mortality, REL: Relapse, aGVHD: Acute Graft-versus-Host Disease, cGVHD: Chronic Graft-versus-Host Disease, CI: Confidence Interval, D100: Day 100, RIC: Reduced-Intensity Conditioning, MAC: Myeloablative Conditioning.

Supplementary Figure 1A

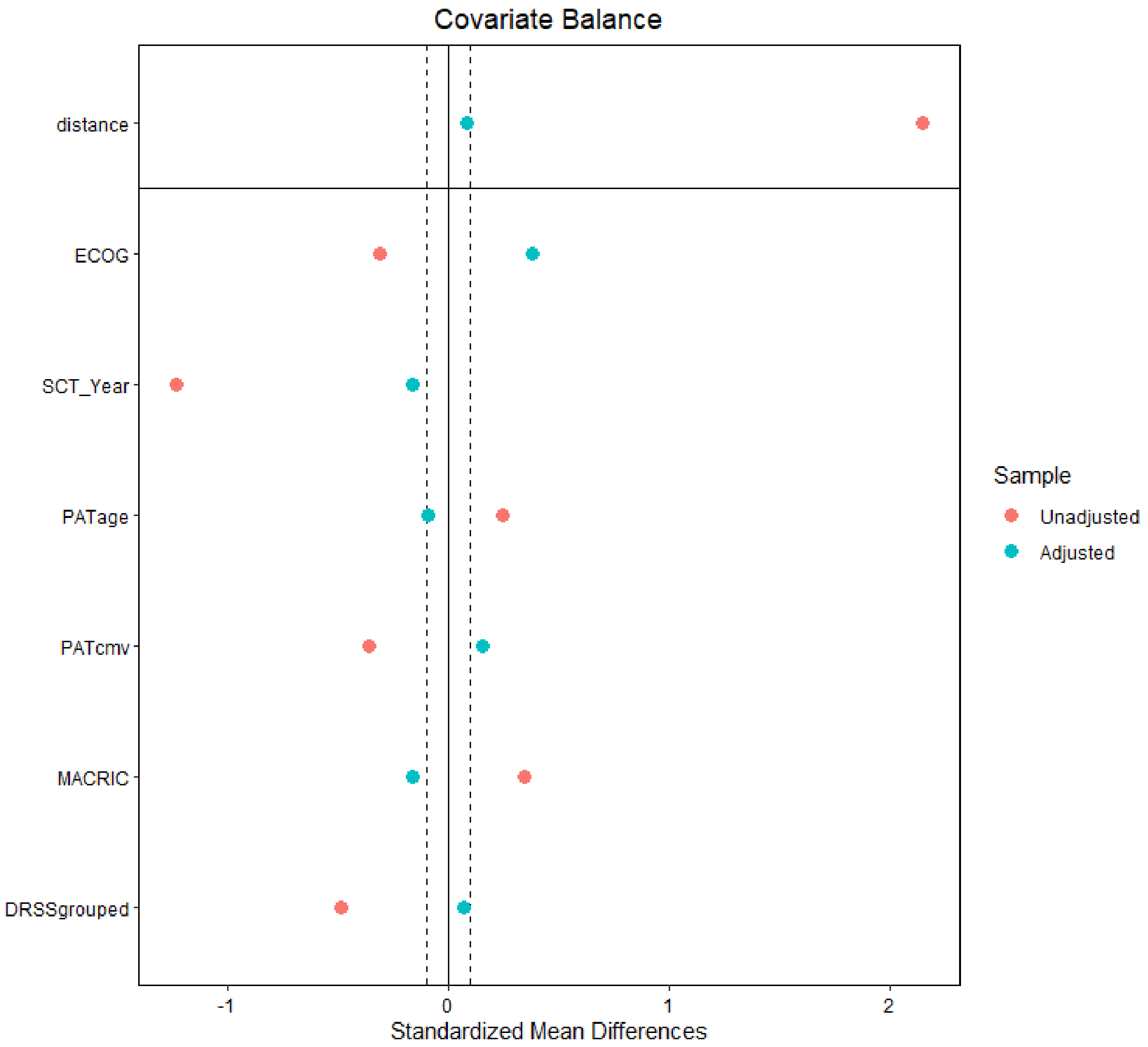

Supplementary Figure 1B

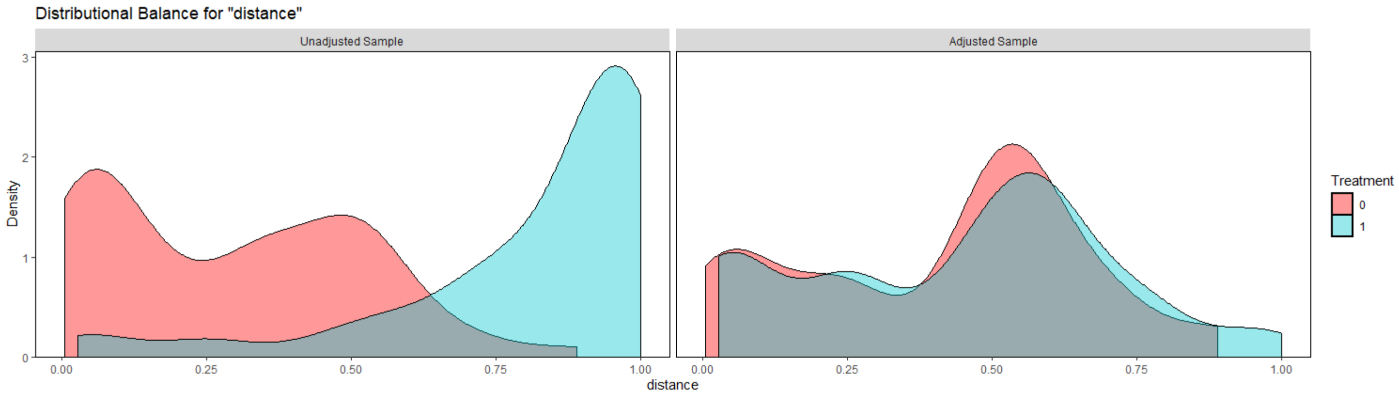

Supplement: Supplementary file 1 — SUpplementary Material [file 41409_2025_2761_MOESM1_ESM.pdf]
